# Delivery of Human iPSC‐Derived RPE Cells in Healthy Minipig Retina Results in Interaction Between Photoreceptors and Transplanted Cells

**DOI:** 10.1002/advs.202412301

**Published:** 2025-04-02

**Authors:** Anna Macečková Brymová, Francisco Javier Rodriguez‐Jimenez, Annika Konrad, Yaroslav Nemesh, Muhammed Arshad Thottappali, Ana Artero‐Castro, Ruslan Nyshchuk, Anastasiia Kolesnikova, Brigitte Müller, Hana Studenovska, Jana Juhasova, Stefan Juhas, Ivona Valekova, Dunja Lukovic, Claudia Aleman, Taras Ardan, Saskia Drutovič, Jan Motlik, Zdenka Ellederova, Zbinek Straňák, Miroslav Veith, Lyubomyr Lytvynchuk, Ruchi Sharma, Kapil Bharti, Goran Petrovski, Pavla Jendelova, Knut Stieger, Slaven Erceg

**Affiliations:** ^1^ Institute of Animal Physiology and Genetics Czech Academy of Sciences Libechov 27721 Czech Republic; ^2^ Department of Cell Biology Faculty of Science Charles University Prague 12800 Czech Republic; ^3^ Stem Cell Therapies in Neurodegenerative Diseases Lab Centro de Investigación Príncipe Felipe Valencia 46012 Spain; ^4^ Department of Ophthalmology Justus‐Liebig‐University Giessen 35390 Giessen Germany; ^5^ Institute of Macromolecular Chemistry Czech Academy of Sciences Prague 16200 Czech Republic; ^6^ Retinal degeneration Lab Department of Animal Production and Health Public Veterinary Health and Food Science and Technology School of Veterinary Medicine Universidad Cardenal Herrera‐CEU, CEU Universities Alfara del Patriarca 46115 Spain; ^7^ 3rd Faculty of Medicine Charles University in Prague, Vinohrady Teaching Hospital Department of Ophthalmology Prague 10034 Czech Republic; ^8^ Ocular and Stem Cell Translational Research Section National Eye Institute NIH Bethesda MD 20892 USA; ^9^ Department of Ophthalmology Center for Eye Research Oslo University Hospital and University of Oslo Oslo 0192 Norway; ^10^ Department of Ophthalmology Faculty of Medicine University of Szeged Szeged 6720 Hungary; ^11^ Institute of Experimental Medicine Czech Academy of Sciences Department of Neuroregeneration Prague 14220 Czech Republic

**Keywords:** age‐related macular degeneration, cell therapy, Human induced pluripotent stem cells;minipigs, retina, retinal degeneration, retinal pigment epithelium

## Abstract

In late stages of inherited and acquired retinal diseases such as Stargardt disease (STGD) or dry age‐related macular degeneration (AMD), loss of retinal pigment epithelia (RPE) cells and subsequently photoreceptors in the macular area result in a dramatic decline of central visual function. Repopulating this area with functional RPE cells may prevent or decline the progression of photoreceptor loss. In the present study, the viability, survival, and integration of human induced pluripotent stem cell (hiPSC)‐derived RPE cells (hiPSC‐RPE) is assessed generated using clinical‐grade protocol and cultured on a clinically relevant scaffold (poly‐L‐lactide‐*co*‐D, L‐lactide, PDLLA) after subretinal implantation in immunosuppressed minipigs for up to 6 weeks. It is shown that transplanted hiPSC‐RPE cells maintain the RPE cell features such as cell polarity, hexagonal shape, and cell–cell contacts, and interact closely with photoreceptor outer segments without signs of gliosis or neuroinflammation throughout the entire period of examination. In addition, an efficient immunosuppressing strategy with a continuous supply of tacrolimus is applied. Continuous verification and improvement of existing protocols are crucial for its translation to the clinic. The results support the use of hiPSC‐RPE on PDLLA scaffold as a cell replacement therapeutic approach for RPE degenerative diseases.

## Graphical abstract

Human induced pluripotent stem cell‐derived retinal pigment epithelial (hiPSC‐RPE) cells, cultured on a clinically relevant PDLLA scaffold, are transplanted into immunosuppressed minipigs. The study demonstrates the viability, integration, and preservation of RPE‐specific features without gliosis or neuroinflammation, supporting their potential as a cell replacement therapy for retinal degenerative diseases.

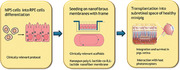

## 1 Background

Stargardt disease (STGD) is the most frequent juvenile maculopathy and age‐related macular degeneration (AMD) is considered the leading cause of vision loss among patients over 60 years.^[^ ^1^, ^2^ ^]^ In both STGD and AMD, the central loss of retinal pigment epithelial (RPE) cells leads to the subsequent degeneration of photoreceptors (PR). This, in turn, results in a significant decline in central visual acuity, posing a severe burden on the quality of life for patients.^[^ ^3^ ^]^ The reason for the early loss of central versus peripheral RPE cells is currently unknown but might be associated with different populations of metabolically active RPE cells in the human retina.^[^ ^4^ ^]^ Progression of these lesions results in continuous loss of RPE cells and subsequent loss of PR, as they lack their physiological counterpart cell.

Repopulating damaged retinae with viable RPE through cell transplantation has the potential to restore functional PR/RPE interfaces, thus preventing or declining the progression of PR loss. This can be achieved by placing new cells into the border zone of the damaged area in STGD and AMD, or by applying them as a patch onto the damaged RPE/Bruch's membrane to close holes in the endogenous RPE monolayer.^[^ ^5^, ^6^ ^]^ Different RPE cell sources have been used as transplants such as primary RPE cells from human cadaveric eyes,^[^ ^7^ ^]^ fetal RPE,^[^ ^8^ ^]^ and derived from pluripotent stem cell sources such as human embryonic stem cells (hESC)^[^ ^9^ ^]^ or induced pluripotent stem cells (hiPSC).^[^ ^9^, ^10^ ^]^ The latter can be generated by reprogramming accessible somatic cells, such as fibroblasts or lymphocytes,^[^ ^11^, ^12^ ^]^ thereby circumventing ethical concerns associated with hESCs. The approach based on hiPSCs can be applied as autologous RPE replacement through the derivation of patient‐specific hiPSC‐RPE cells.^[^ ^6^ ^]^ Alternatively, RPE cells derived from a master bank of deposited healthy hiPSCs can be employed for allogeneic therapy.^[^ ^13^ ^]^ In both scenarios, the development of an efficient differentiation protocol is crucial to generate a monolayer of pure, highly polarized RPE cells devoid of undifferentiated hiPSCs in transplanted material, thereby mitigating the risk of teratoma formation.

Pluripotent stem cells, such as hESCs and hiPSC have been subjected to diverse protocols that involve 2D or culture 3D systems or co‐culturing with supporting cells to enhance the maturation and functionality of derived RPE cells (reviewed by Artero‐Castro et al.^[^ ^14^ ^]^). While most of the approaches yield RPE‐like structural and functional features, constant optimization is mandatory to assure cell identity, purity, and scalable production. For therapeutic applications it is also crucial to assess the surgical transplantation method involving surgical devices^[^ ^13^, ^15^, ^16^ ^]^ and the use of immunosuppression regimes as these are often contributing factors to adverse events in vivo.

In this regard, transplantations in large animal models are essential due to anatomic similarities to the human eye. Moreover, the ability to apply techniques adopted in the human clinical practice to assess the morphology and function of the retina such as OCT and ERG accelerates the path of tested therapies toward the clinic. Consequently, the pig has emerged as a pivotal preclinical model for investigating the transplantation of RPE cells.^[^ ^6^, ^17^ ^]^

Bharti et al. introduced an innovative protocol for differentiating hiPSCs into RPE tailored for clinical applications.^[^ ^6^, ^18^ ^]^ The advantages of this approach involve high reproducibility between cell lines, high purity, and efficacy of differentiation while reducing the overall duration of the protocol compared to spontaneous differentiation‐based approaches.^[^ ^6^, ^19^, ^20^ ^]^ HiPSC‐RPE cells were seeded on nanofibrous scaffolds which are proposed to support extracellular matrix (ECM) secretion and cell polarization, in addition to aiding surgical handling and maintenance of the cell organization as monolayer during transplantation.^[^ ^21^ ^]^ In the realm of RPE cell culture, the choice of scaffold material plays a pivotal role in nurturing cell growth and maintaining physiological functionality. Among the array of scaffold options, polylactic‐*co*‐ glycolic acid (PLGA) and polylactic acid (PLA) derived membranes emerge as promising candidates due to their unique blend of biocompatibility and biodegradability. These polymer‐based scaffolds provide a supportive matrix for RPE cell adhesion, proliferation, and differentiation, mimicking the natural extracellular environment. Notably, their inherent biodegradability over time aligns with the dynamic nature of cellular processes, ensuring seamless integration and tissue regeneration.^[^ ^22^, ^23^ ^]^ In this work, we produced hiPSC‐RPE cells according to the above‐mentioned protocol as patches using nanofibrous PDLLA membranes and delivered such patches subretinally into a healthy minipig eye. The histological analysis was performed at 1, 2, and 6 weeks post transplantation to observe structural integration, alterations in retinal structure, tumor or ectopic tissue formation, and signs of toxicity and inflammation.

## 2 Results

### 2.1 Generation of RPE Cells from hiPSCs Derived from Healthy Individuals Using a Clinical‐Grade Triphasic Protocol

**Figure 1:**
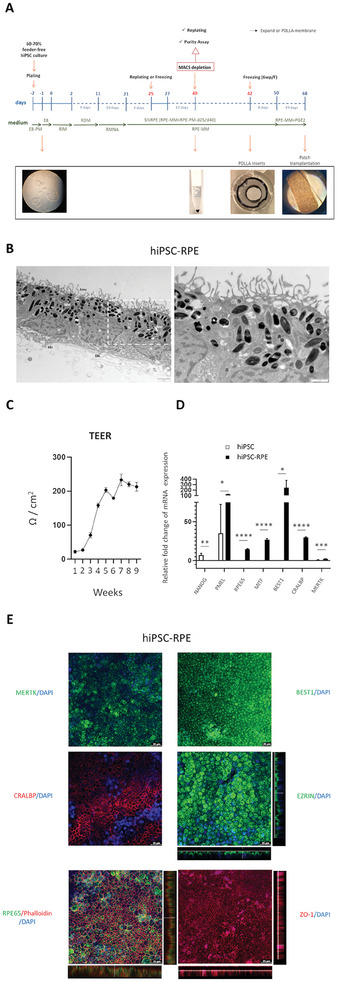
Differentiation of hiPSCs into RPE. A) Timeline of clinical‐grade RPE differentiation. Clinical‐grade RPE differentiation protocol begins using a hiPSC monolayer and uses clinical‐grade reagents. PM, plating medium; RIM, retinal induction medium; RDM, retinal differentiation medium; RMNA, retinal medium with Nicotinamide and Activin‐A; RPE‐MM, RPE‐maintenance medium. Lower images, Representative brightfield micrographs with hiPSC at the beginning of the protocol, the insert with the oval frame in the center, and higher magnification of the insert with highly pigmented cells at the time of transplantation B) Representative electron micrograph of cultured hiPSC‐RPE on porous scaffold. Apical microvilli (AmV), melanosomes (Mel), tight junction (white arrows), and basal infoldings (SBI) are detected. The nuclei (N) are located on the basal side of the cells. C) TEER values of hiPSC‐RPE patches during in vitro cell culture. D) qRT‐PCR analysis of mRNA expression for RPE markers (PMEL, RPE65, MITF, BEST1, CRALBP, MERTK) and the pluripotency marker NANOG. Data represents the fold‐change in mRNA expression of hiPSC‐ RPE relative to their parental hiPSCs. Each bar represents the average ± SEM of at least three independent replicates. ^**^ *p* ≤ 0.01, ^***^ *p* ≤ 0.001, ^****^ *p* ≤ 0.0001. E) Immunocytochemical analysis of RPE marker protein expression (MERTK, BEST1, CRALBP, EZRIN, RPE65 y ZO‐1) in hiPSC‐RPE patches. Vertical confocal sections showing apical localization of EZRIN and ZO1. Images were taken with Leica confocal microscope TCS SP8 using HCX PL APO lambda blue 63X/ 1.4 OIL objective. Scale bar = 25 µm.

To establish a viable cell source for replacement therapy, it is imperative to implement clinically compliant culture conditions for the differentiation of hiPSCs derived from healthy individuals. In this investigation, we utilized a hiPSC line previously developed within our research group obtained from a healthy donor (Ctrl3‐FiPS4F1),^[^ ^24^ ^]^ applying a GMP‐compliant and defined conditions to induce RPE cell fate as outlined by Sharma et al.^[^ ^6^, ^18^ ^]^ (**Figure** **1**A). This protocol is rooted in earlier investigations that induced neuroectoderm differentiation of hiPSCs by dual‐SMAD inhibition and induced RPE differentiation by activating the ACTIVIN A signaling.^[^ ^25^, ^26^, ^27^ ^]^ On day 40 of the protocol, we separated immature hiPSC‐RPE from non‐RPE cells by negative magnetic immunoreactivity (CD24^−^ and CD56^−^) as previously described.^[^ ^6^, ^18^ ^]^ To achieve the full maturation of hiPSC‐RPE, the cells were seeded onto an electrospun PDLLA biodegradable membrane. This scaffold comprises a biodegradable ultrathin nanofibrous membrane crafted from PDLLA.^[^ ^17^ ^]^ The inclusion of a supporting oval polyethylene terephthalate (PET) frame (5.2 mm × 2.1 mm) into the membrane was required to provide mechanical resistance during the excision of the patch from the plastic insert and throughout surgical procedures (Figure 1A). The maturation of hiPSC‐RPE was followed by observation of pigmentation intensity, acquisition of polygonal form, and transepithelial/transendothelial electrical resistance (TEER) measurements (Figure 1C). At this time point, some of the membranes were separated and subjected to characterization while others were prepared for transplantation.

Ultrastructural studies using transmission electron microscopy (TEM) confirmed the correct apical location of microvilli and lateral tight junctions between neighboring cells with cell nuclei located basally six weeks after plating the hiPSC‐RPE on the nanofibrous membrane (Figure 1B). Ellipsoidal mitochondria were detected below the nuclei at the basolateral side of RPE while melanosomes were present apically. The hexagonal morphology and high level of pigmentation were acquired at this stage, in appropriately aligned sections in RPE patches. Basal infoldings toward nanofibrous scaffolds were also observed (Figure 1B). All hiPSC‐RPE‐patches demonstrated progressively increasing TEER suggesting gradual acquisition of maturity. The TEER values reached 200 Ω cm^2^ upon 6 weeks (transplantation time point) of culture which suggests the intact epithelial cell layer with established tight junctions (Figure 1C).

To assess the quality of the hiPSC‐RPE patches, we conducted thorough quality check characterizations. Gene expression analysis for trait RPE markers demonstrated significantly higher expression levels of *PMEL*, *RPE65*, *MITF*, *BEST1*, *CRALBP*, and *MERTK*, compared to respective undifferentiated hiPSC (Figure 1D). The gene expression analysis further validated the absence of pluripotency‐associated marker *NANOG* in hiPSC‐RPE cells (Figure 1D) consistent with the notion that hiPSCs have acquired RPE cell fate.

Immunocytochemical analysis revealed the expression of MERTK, BEST1, CRALBP, RPE65, and the microvilli marker EZRIN (expressed apically) (Figure 1E). The positive staining for the tight junction marker zonula occludens protein‐1 (ZO‐1) provides qualitative insights into the epithelial monolayer barrier integrity (Figure 1E).

### 2.2 Implantation of the hiPSC‐Derived RPE Patches

After culturing the patches for 6 weeks on PDLLA scaffolds, the membranes were released from the insert and the PET frame embedded in the membrane area of 5.2 mm × 2.1 mm was excised by a shaped biopsy punch before loading into the implantation injector. Recently, we described a safe and effective procedure to subretinally implant human primary RPE (hRPE) cells in minipig eyes.^[^ ^17^ ^]^ More than 90% of all 29 surgeries (in 18 animals) resulted in efficient integration.^[^ ^15^, ^17^, ^28^ ^]^ In the current study, the same surgical procedure was used with similar efficiency.

**Figure 2:**
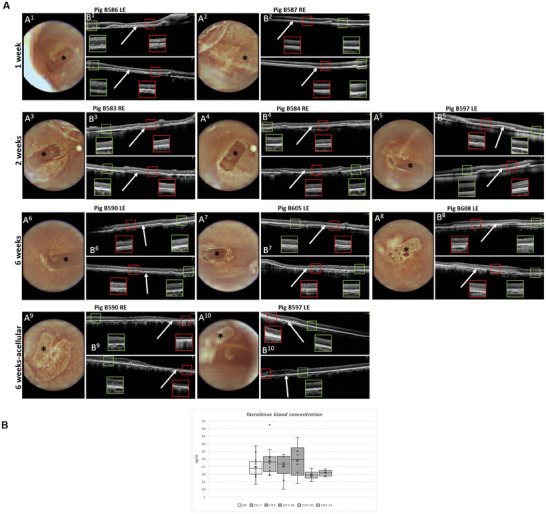
Fundus imaging and OCT of implanted minipig retinas at 1, 2 or 6 weeks post‐implantation. A) A1‐A10, Fundus images of implanted minipig retinas. The ^*^ indicates the position of the implant in the eye fundus. B1‐B10, Fundus‐paired black‐white OCT scans (upper scans are horizontal, lower scans are vertical) of implanted retinas. White arrows show the location of the implant in the subretinal space. Magnifications with red borders demonstrate implanted areas of the retina, with green border non‐implanted adjacent areas. Images A9, B9, and A10, B10 demonstrate implanted acellular scaffolds at 6 weeks post‐implantation. Severe retinal atrophy was detected in the part of the neuroretina lying above the scaffolds (B9). The animal is identified by the number preceded by the B letter. Left eye (LE), right eye (RE). B) Tacrolimus concentration in vivo. Time course of intravenous Tacrolimus concentrations in treated animals. D0 – Cell seeded scaffold implantation + Depomedrol 80 mg/pig i.m. application, D5‐7–2nd TLPM s.c. application (TAC dose 0.3–0.8 mg kg^−1^ BW), D14 – termination of animals for 2 weeks’ time point, D17‐19–3rd TLPM s.c. application (TAC dose 0.4–0.5 mg kg^−1^ BW), D29‐30–4th TLPM s.c. application (TAC dose 0.5–0.6 mg kg^−1^ BW), D42‐43–Experiment termination.

Similarly, in this study, the fundus imaging revealed the position of the implanted patch confirming the successful subretinal implantation in the nasal area of the retina (Figure 2AA^1^‐A^10^). Retinal imaging by OCT showed the optimal implant's adherence to surrounding retinal structures, displaying hyperreflectivity of implanted hiPSC‐RPE patches comparable to native porcine RPE layer, and affirming the precise placement of implants in the subretinal space. OCT scans demonstrated a healthy neural retina with preserved inner and outer retinal layers with a minimal decrease of retinal thickness at the implant site, exhibiting complete healing of the retinotomy without a proliferative reaction to the implant (Figure 2AB^1^‐B^8^). However, in the case of implantation of acellular scaffolds, severe retinal atrophy was observed in the place of the implantation (Figure 2AB^9^).

### 2.3 Immunosuppression and Analysis of Inflammation

Starting one week before surgery and throughout the experimental timeline, immunosuppression was performed using repeated subcutaneous tacrolimus‐loaded polymer microspheres (TLPM) injections. Tacrolimus levels in serum were measured every week and were shown to be above the level that was shown to be the minimal effective level (Figure 2B). Our findings reveal that using this immunosuppression method we have maintained a consistent tacrolimus concentration in porcine blood. Furthermore, cytokine levels of pro‐inflammatory cytokines were repeatedly measured in the plasma of the transplanted pigs, but no elevated values were detected (Figure S1, Supporting Information).

### 2.4 Immunohistochemical Analysis of the Implanted hiPSC‐RPEs

**Figure 3:**
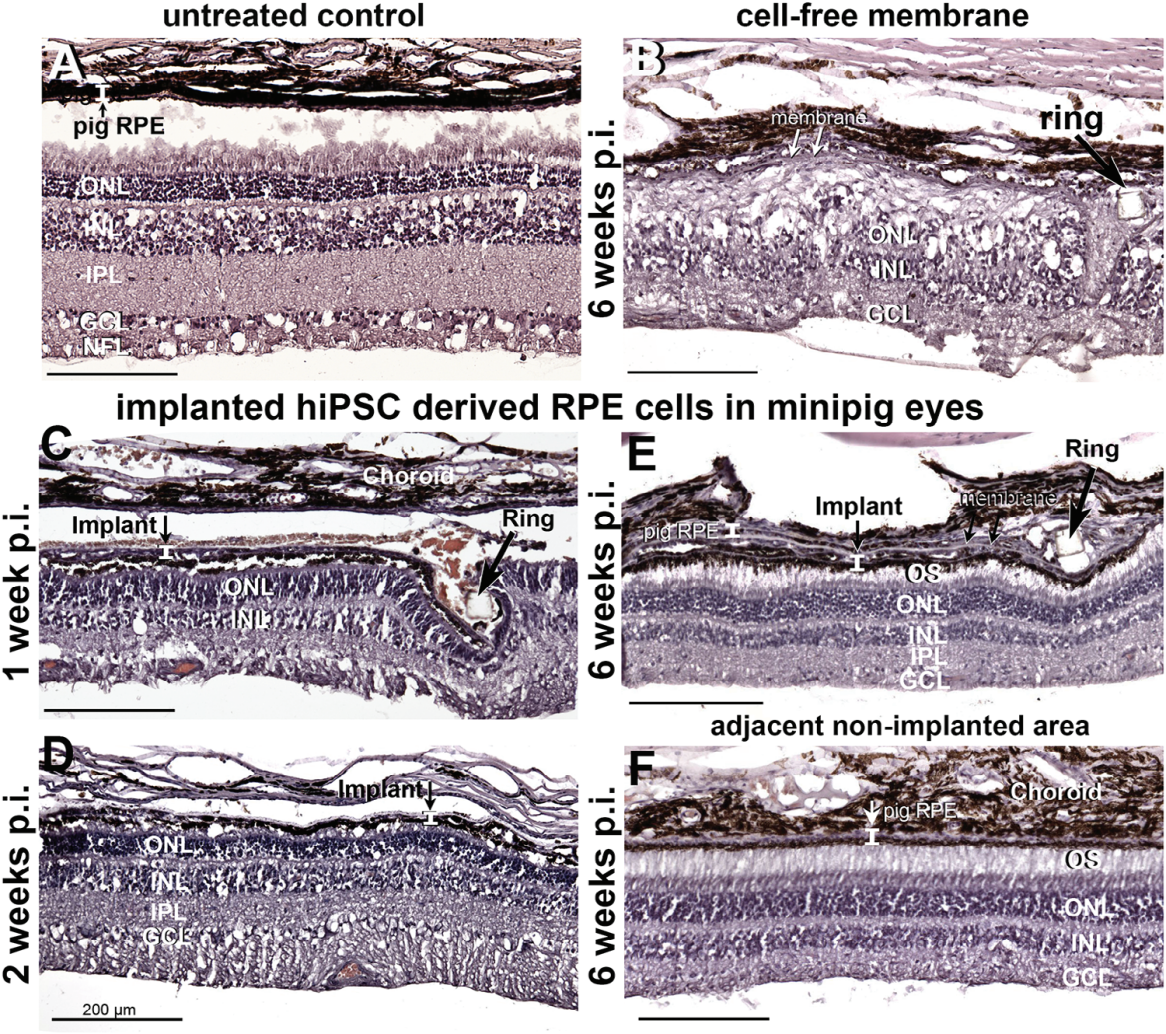
Hematoxylin & eosin staining of the retinal area containing the transplanted cells on nanofibrous carrier membrane surrounded by PET frame structure (arrows). Implanted hiPSC‐derived RPE cells are heavily pigmented and in close proximity to photoreceptor inner and outer segments. Observations are presented at 1 (C), 2 (D), and 6 (E) weeks post‐implantation as well as an untreated control eye (A). The level of the implanted RPE cell layer is marked with a bar indicating the heights of the RPE cells (marked by a medium sized arrow in C–E). Retina with implanted cell‐free membrane shows a disorganized outer nuclear layer after six weeks (B). Big arrows in B, C & E point to the PET frame (ring) of the PDLLA nanofibrous membrane. The retina adjacent to the implanted area is unaffected by the implantation F). Carrier dimensions, 5.2 mm × 2.1 mm. ONL = outer nuclear layer; INL = inner nuclear layer; IPL = inner plexiform layer; GCL = ganglion cell layer; NFL = nerve fiber layer. All scale bars represent 200 µm.

**Figure 4:**
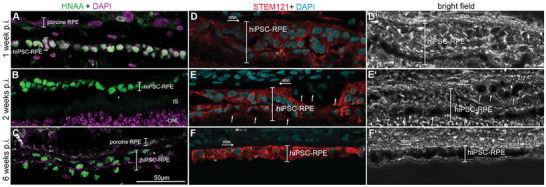
Expression of Human Nuclear Antigen (HNAA, green) and STEM121 (red) on hiPSC‐RPEs implanted on nanofibrous carriers, followed up to 6 weeks. Tissue was stained with HNAA A–C) and STEM121 D–F) antibodies. The third panel (D’‐F’) shows bright field images of the STEM121 staining. Observations are presented at 1 (A, D, D’), 2 (B, E, E’), and 6 (C, F, F’) weeks. Arrows in E and E’ point to heavily pigmented RPE cells partially masking immunohistochemical staining in implanted hiPSC‐RPE cells. They appear as a monolayered or a multi‐layered cell structure after 1, 2, or 6 weeks showing a high cell density., i.e., the distance between the nuclei is very small which is unusual for RPE cells. Nuclear staining was performed by DAPI (purple and light blue). ONL = outer nuclear layer; nfm = nanofibrous membrane; hiPSC‐RPE = human iPSC derived RPE cells; RPE = retinal pigment epithelium.

**Figure 5:**
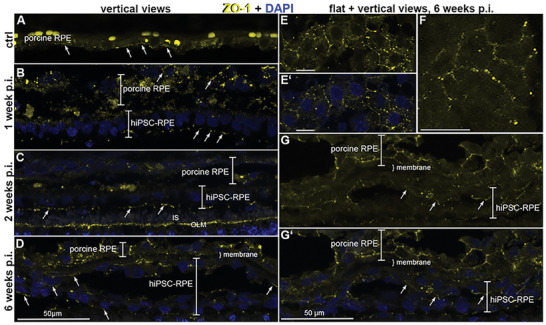
Immunohistochemical detection of tight junction marker ZO‐1 (red). Vertical and flat sections through the posterior part of the minipig eye holding implanted hiPSC‐RPE cells on nanofibrous carrier membrane after 1 (B) 2 (C) and 6 weeks (D–G). In flat views, the typical hexagonal shape of RPE cells is nicely visible (E, E’, F) due to the ZO‐1 immunoreactive staining of the tight junctions. In vertical views, the ZO‐1 staining appears rather dot‐like or like a dashed line (arrows in A–D, G, G’). Nuclear staining by DAPI is shown in magenta. OLM = outer limiting membrane. The scale bar in D holds for A–D, in G‘ for G, scale bar in E‐F, 10 µm.

At one, two, and six weeks post‐implantation (three pigs per time point, Figures S2 and S3, Supporting Information) pigs were euthanized, the eyes enucleated, and morphological analysis by immunohistochemistry was performed. The nanofibrous membrane with the hiPSC‐RPE cells was easily identified in the fundus of the pig eye at all time points (**Figures** **3**,4, 5; Figures S2 and S3, Supporting Information).

Implanted hiPSC‐RPE cells were heavily pigmented and observed in close proximity to photoreceptor inner and outer segments (Figure 3C–E). Due to dissection procedures, tissue layers were separated in some areas and represent manipulation artifacts. Retinal integrity underneath the implanted patch improved with post‐implantation time. After six weeks the retinal layers appeared preserved and similar to the untreated control eye (Figure 3E). Minipig retina with implanted cell‐free membrane shows a disorganized outer nuclear layer after six weeks (Figures 3B, 4C,D,H‐I’). The retina adjacent to the implanted area is unaffected by the implantation (Figure 3F).

Immunoreactivity to human markers such as Human Nuclear Antigen (HNAA) and STEM121 was observed in hiPSC‐ RPE cells at all time points investigated (Figure 4; Figure S3, Supporting Information). Interestingly, both stainings revealed cells displayed as monolayers in addition to multilayered cell sheets. The cell multilayering seemed to be independent of the time elapsed after implantation. Moreover, we observed both the mono‐layered and multi‐layered hiPSC‐RPE cells along the same implanted nanofibrous membrane in almost all samples. At one and two weeks after implantation, staining with human cell markers showed hiPSC‐RPE cells densely packed revealing a short distance between nuclei which is unusual for RPE cells. However, six weeks after implantation distance between hiPSC‐RPE cells appeared to be larger (Figure 4C,F; Figure S3G,I,P,R, Supporting Information). Mostly when arranged in a monolayer the hiPSC‐RPE nuclei are well organized and hence more RPE‐like appearance (Figure 4F; Figure S3P,R, Supporting Information).

In addition, heavily pigmented cells partially hindered immunohistochemical signal in implanted hiPSC‐RPE cells (arrows in Figure 4E,E’).

We found both RPE cell markers, Bestrophin and CRALBP in minipig RPE and implanted hiPSC‐ RPE cells at all time points investigated (Figure S4A–H, Supporting Information). The expression of Bestrophin seemed highest at 6 weeks post‐implantation. CRALBP immunofluorescence appeared to be less prominent in implanted hiPSC‐RPE cells compared to endogenous pig RPE cells at all time points investigated (Figure S4E–H, Supporting Information).

Immunoreactivity to the tight junction marker ZO‐1 was observed in hiPSC‐RPE cells as well as in pig endogenous cells at all time points investigated (**Figure** **5**A–D,G). Notably in vertical sections of the minipig retina, the tight junctions appear as signals located apically in the implanted hiPSC‐RPE cells and host porcine RPE cells (arrows in Figure 5A–D). In flat views, the typical hexagonal shape of RPE cells is appreciated in both the porcine RPE cells and the implanted hiPSC‐RPE cells. Especially after six weeks, the tight junctions seem to be well preserved in the human and the minipig RPE cells (Figure 5E–G’). In addition, tight junctions between Müller glia cell endfeet and the inner segments of photoreceptor cells are brightly labeled by the ZO‐1 antibody (Figure 5C), corresponding nicely with the location of the outer limiting membrane (OLM).

**Figure 6:**
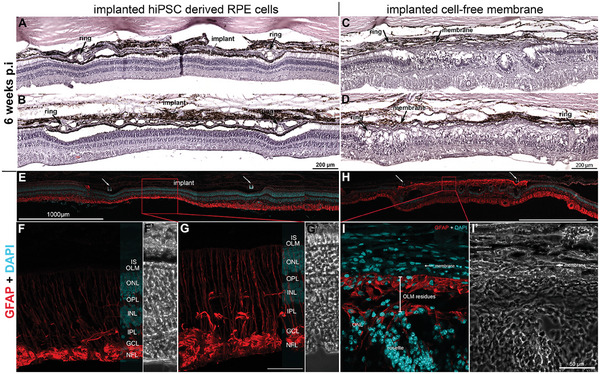
Hematoxylin & eosin staining of the retinal area containing the implanted nanofibrous carrier membrane with and without hiPSC‐RPE cells A–D). Massive rosette formation underneath the cell‐free nanofibrous membrane is visible (C, D, I). In contrast, no rosette formation was detected in the retina underneath the implanted carrier membrane with the human RPE cells (A, B, E). Immunohistochemistry of reactive gliosis marker GFAP in minpig retina showed massive reactive gliosis after implantation of cell‐free nanofibrous carrier membrane after six weeks (H, I). No signs of reactive gliosis or rosette formation were detected in the retina underneath the implanted carrier membrane with the human RPE cells (A, B, E–G). Massive growth of Müller glia cell processes is visibly filling the gap of the dying photoreceptors (H‐I’). (F’, G’, I’) represent the respective bright field images of (F, G, I). Nuclear staining by DAPI is shown in blue. Arrows mark the PET ring structure surrounding the nanofibrous carrier membrane (A–D, E, H). IS = inner segment, OLM = outer limiting membrane, ONL = outer nuclear layer, OPL = outer plexiform layer, INL = inner nuclear layer, IPL = inner plexiform layer, GCL = ganglion cell layer, NFL = nerve fiber layer. Scale bar in G, 50 µm.

In H&E stained retinal sections no histological signs of rosette formation in the photoreceptor layer or reactive gliosis are visible in the retina underneath the implanted carrier membrane with the hiPSC‐RPE cells (**Figure** **6**A,B,E–G). In contrast, six weeks after implantation of cell‐free nanofibrous carrier membrane we did observe massive gliosis using reactive gliosis marker GFAP in addition to rosette formation visible in H&E stainings (Figure 6C,D,H,I), indicating massive neurodegeneration at the transplantation site.

**Figure 7:**
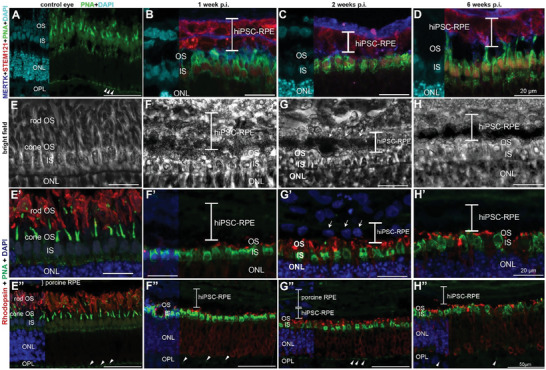
Expression of peanut agglutinin (PNA, green), MERTK (blue), and STEM121 (red) in the upper row B–D) and PNA and rhodopsin (red) (E’‐H’, E” – H”) in treated and untreated minipig eyes (the two lower rows). The second row (E–H) represents the bright field images of the respective time points and images in the two lower rows. Nuclear staining was performed by DAPI (light blue or dark blue). Observations are presented at 1 (B, F, F’, F”), 2 (C, G, G’, G”), and 6 (D, H, H’, H”) weeks post‐implantation as well as an untreated control eye (A, E, E’, E”). PNA brightly labels the inner and outer segments of cone photoreceptors in treated eyes. Cone pedicles are labeled by PNA too but less intensely (arrowheads). MERTK is localized in the microvilli of RPE cells. Especially at 6 weeks p.i. Close connections are visible between PNA‐positive cone photoreceptor outer segments and MERTK‐immunoreactive microvilli of hiPSC‐RPE cells (D). In one animal each at week 1 p.i. and week 2 p.i. We observed a close connection between cone outer segments and hiPSC‐RPE microvilli. Rod outer segments labeled by the rhodopsin antibody are present to some extent at all time points shown here (F’‐H’, F”–H”). Bright‐field images of the retinal implant region reveal heavy pigmentation of the hiPSC derived RPE cells and their close proximity to photoreceptor outer segments (F–H). hiPSC‐RPE = human iPSC derived RPE cells; RPE = retinal pigment epithelium, OS = outer segment, IS = inner segment, ONL = outer nuclear layer, OPL = outer plexiform layer. The scale bar in A, E”‐H” is 50 µm. Scale bars in B‐D, E‐H, E’‐H’ are 20 µm.

In order to further study potential interactions between endogenous porcine PR and transplanted human RPE cells, the connections between cone outer segments (COS) and hiPSC‐RPE cell microvilli were analyzed using Peanut Agglutinin (PNA) and MERTK staining (**Figure** **7**B–D). Co‐labeling with PNA and Rhodopsin antibody revealed the condition of all PR outer and inner segments in control and treated minipig retina (Figure 7E″–H″). In untreated minipig retina, PNA binds strongly in the COS and moderately in their synaptic pedicles (Figure 7A, arrowheads in E″). In the untreated control retina, the inner segments of cones (CIS) show hardly any fluorescence. One and two weeks after transplantation, the PNA binding pattern looks different, since the outer segments of PR are quite short and CIS appears swollen and oval due to the short‐term separation from the porcine RPE during surgery. Now, residues of COS and the plasma membrane of the CIS show high fluorescent PNA binding (Figure 7B,C,F'G’).^[^ ^27^ ^]^ Cone pedicles show some PNA binding as well 1 and 2 weeks post transplantation but less intensely compared to control eyes (Figure 7F″,G″, arrowheads). At 6 weeks post transplantation, PNA‐stained COS and MERTK‐immunoreactive microvilli of hiPSC‐RPE cells are localized in close proximity (Figure 7D). In one animal, this close connection between porcine COS and human RPE cell microvilli was observed even at week 1 and 2 p.i. each Figure (7B). Bright‐field images of the retinal implant region corroborate the notion that photoreceptor outer segments and hiPSC‐derived RPE cell implants are positioned in close proximity (Figure 7F–H). Short rod outer segments labeled by the rhodopsin antibody are present at all time points investigated in this study (Figure 7F’–H’). We also note that rhodopsin appears delocalized into the photoreceptor perikaryal (Figure 7F″–H″) which is likely caused by the stress of separation from the native RPE layer.

Staining with the microglia marker Iba1 reveals microglial cells and their processes at all time points investigated (Figure S5, Supporting Information). There appears to be a minimal disparity in iba1‐immunoreactivity between the transplanted area and regions outside (Figure S5B–G, Supporting Information). Notably, one‐week post‐transplantation, cell bodies of Iba1‐immunoreactive microglial cells are not readily observable (Figure S5B,E, Supporting Information). In the inner plexiform and ganglion cell layers, only dot‐like rough processes are notably evident. Two weeks post transplantation, individual microglial somata exhibit Iba1 immunoreactivity (Figure S5C,F, Supporting Information, indicated by arrows). However, outside the implant area, more microglial cells and their processes are detectable. By six weeks post‐transplantation, Iba1‐immunoreactive cells are mainly present in the INL and IPL, with their processes branching into the nuclear and plexiform layers in a characteristic manner, akin to untreated minipig eyes (Figure S5A,D,G, Supporting Information). It is noteworthy to highlight that we did not observe any teratoma formation during the 6 weeks of in vivo analysis.

## 3 Discussion

In this study, we show the successful transplantation of scaffold based hiPSC derived RPE cells into the subretinal space of minipigs for up to 6 weeks concurrent with first evidence of continuous epithelial phenotype preservation and interaction with adjacent photoreceptors. Surprisingly, in certain areas of the scaffolds, multilayering of RPE cells occurred without evidence of proliferative activities. Transplantation of cell free scaffolds led to massive degeneration of overlying retinal cells, indicating a barrier function of the scaffold in the absence of functional RPE cells, further corroborating the notion that the transplanted hiPSC‐RPE cells served as interaction partners with endogenous porcine cells.

For allogenic and autologous cell therapy to be successful, cell manufacturing processes that are both efficient and reproducible, ultimately yielding a safe and effective product, are mandatory. Survival of cells and functional integration into the retinal area are the subsequent critical steps toward clinical use. Furthermore, many unknown parameters, such as iatrogenic aspects and neuroinflammatory response to the transplant render it difficult to estimate success rates. As a novelty in this study, we have investigated the survival and integration of hiPSC‐RPE cells matured on electrospun PDLLA scaffold after transplantation into wild‐type porcine retina. The hiPSCs‐RPE cells were generated according to the clinically compliant protocol on PDLLA nanofibrous scaffold extending its applicability beyond PLGA membranes described previously.^[^ ^6^ ^]^ Compared to PLGA scaffolds, which degrade within three months and risk structural weakening during transplantation,^[^ ^6^ ^]^ PDLLA carrier offers superior mechanical stability. Ultrathin PDLLA membranes (≤4 µm), used in our study, are less biodegradable, maintaining structural integrity for 6 months to 2 years.^[^ ^29^ ^]^ This durability ensures functional support throughout transplantation and early integration. Key design enhancements include a supporting frame that improves handling, allows precise graft loading, and prevents damage by isolating the cell layer from direct injector contact. Orientation markers further aid implantation accuracy.^[^ ^17^ ^]^ These features enable a reproducible surgical success rate of 93.1%, with failures unrelated to the carriers.^[^ ^15^ ^]^ RPEs cultured on PDLLA nanofibers formed high‐quality epithelial monolayers with better differentiation, phagocytic activity, confluence, and long‐term viability than those on commercial track‐etched membranes.^[^ ^30^ ^]^ The PDLLA scaffold was selected not only for its specific biodegradability but also for its favorable biocompatibility and low immunogenicity.^[^ ^31^ ^]^ The described biodegradability time of a few months confers an advantage as it allows the transplanted cells to produce their own Bruch's membrane components and additionally avoids the formation of fibrosis observed when using nonbiodegradable scaffolds.^[^ ^19^, ^32^ ^]^

The hiPSC‐RPE patches used in this study contain a fully‐polarized monolayer of cells that express main RPE markers (*PMEL, RPE65, MITF, BEST1, CRALBP, MERTK*). The high TEER values reached concurrently in multiple inserts at 6 weeks of culture on nanofibrous membrane indicate mature tight junctions which is a crucial property of the RPE cells and required for maintenance of the outer blood‐retinal barrier. Furthermore, IHC staining confirmed the expression of epithelial tight junction marker ZO‐1, providing evidence for the epithelial phenotype of the cells on the scaffold. In conclusion, cultured hiPSC‐RPE cells described here showed the amenability to be induced with similar efficiency to the originally described protocol.^[^ ^18^ ^]^

PDLLA scaffold with cells remained intact and stable for at least 6 weeks in vivo. This integration seems to be a result of a coordinated process involving slow degradation of the PDLLA scaffold and the possible concurrent production of extracellular matrix (ECM) by hiPSC‐RPE. This orchestrated mechanism may facilitate the seamless integration of hiPSC‐RPE with the host in subsequent studies involving animal models with RPE damage at the target site. Our findings indicate that hiPSC‐RPE cells not only survive but also demonstrate sustained integration within the host tissue, persisting for at least six weeks in minipig subretinal space. On the other hand, our double bent injector contributed to the successful patch implantation by not causing RPE damage.

In this study, tacrolimus was applied as an immunosuppressive agent. As a calcineurin inhibitor, tacrolimus inhibits the binding of T cell activation by antigen‐presenting cells (APCs) and also inhibits the presentation of antigen by class I‐ and class II‐restricted APCs to T cells.^[^ ^33^, ^34^ ^]^ In the context of cell transplantation, ensuring a continuous release of tacrolimus into the bloodstream is crucial for maintaining effective immunosuppression over an extended period. This continuous supply plays a pivotal role in supporting the success and longevity of cell transplantation by safeguarding against immune responses that could jeopardize the survival and function of the transplanted cells. Maintaining a consistent tacrolimus concentration in pig blood through the use of tacrolimus‐loaded polymer microspheres (TLPM) demands careful consideration. Our previous experience (data not shown) reveals that younger animals with lower body weight necessitate higher TLPM doses. Consequently, the initial tacrolimus dose in TLPM must be thoughtfully selected. In our current approach, we set the first dose at 0.8 mg kg^−1^ body weight, administering it 5 to 7 days before the transplantation of hiPSC‐RPE cells. Depending on the tacrolimus concentration in the blood on day 12, adjustments within the range of 0.3–0.8 mg kg^−1^ body weight in TLPM were made for each pig to maintain the tacrolimus blood concentration between 20 and 30 ng mL^−1^. Successful attainment of this objective, as evidenced by positive study results, affirms that the applied immunosuppression had no adverse effects on the animals and did not pose toxicity to the transplanted human cells.

In contrast, a prior study employing the same TLPM with a single application six days before transplantation proved insufficient. Signs of graft rejection accompanied by neuroinflammation were evident at 6 and 8 weeks.^[^ ^17^ ^]^ Likewise, in a different study, short‐term local application of corticosteroids failed to rescue allogeneic hiPSC‐RPE cells in non‐human primates after subretinal implantation at 3 weeks.^[^ ^35^ ^]^ In summary, maintaining a stable tacrolimus concentration using TLPM in pig blood, with dose adjustments based on individual factors, proved effective in preventing adverse effects or toxicity in transplanted hiPSC‐RPE patches, as demonstrated in this study. These results emphasize the importance of continuous and tailored immunosuppression for successful cell transplantation outcomes.

We were able for the first time to show that ZO‐1 staining was positive and transplanted cells formed tight junctions 6 weeks after surgery. This means that the cells kept their polarity and epithelial character, indicating that viability was granted and the surrounding tissue enabled functional interaction with other cells. The direct interaction was difficult to observe since the transplanted RPE cells were heavily pigmented. Nonetheless, increasing the length of correctly formed outer segments over the duration of the study is a strong indicator of such interaction.

Encouragingly, our observations revealed distinct indications of human–porcine PR interaction, sustained polarity of RPE cells, and an absence of inflammation or glial scar formation.

Indeed, when utilizing the scaffold without RPE cells, severe retinal degeneration was evident, emphasizing the effect of separating photorceptor outer segments from their RPE cells and the barrier function of the scaffold. Furthermore, the presence of hiPSC‐RPE cells demonstrated their pivotal role in fostering RPE‐PR interaction, highlighting the critical importance of the transplantation approach in preventing retinal degeneration. A further indication of the formation of functional RPE‐PR interfaces was the observation that cell‐free scaffolds seem to form a diffusion barrier, as photoreceptor cells degenerate in the absence of hiPSC‐RPE cells. This indirect proof is crucial for subsequent studies, where the presence of the scaffold needs to be analyzed at later time points.

However, the multilayered organization of transplanted RPE cells was detected in most animals despite their monolayer organization on the membranes during in vitro cultures. Several factors may contribute to this intriguing observation. One possibility considered was that some cells adhered to the bottom side of the scaffold during in vitro cell culture, resulting in a double cell layer in the recipient tissue. The low thickness and high porosity due to degradation of our nanofibrous membranes in vitro and in vivo could lie behind this misarrangement. However, we consistently observed the cellular organization on the scaffold at the time of transplantation, and no cells were detected on the bottom side.

Another hypothesis explored was the potential dedifferentiation of hiPSC‐RPE cells during the transplantation period, leading to mitotic activity and subsequent multilayering. However, staining for a proliferation marker (Ki65) yielded negative results, indicating that the cells did not exhibit significant mitotic activity (data not shown). Further investigation is required to fully elucidate the mechanisms underlying this process.

Finally, surgery‐related factors might have caused changes to the monolayer structure on the scaffold. For example, the scaffold is forwarded by a double bent injector underneath the retina, and during this manoever, cells might have been pushed in two or three layers over each other. This might explain the absence of proliferation in the transplanted area despite evident multilayering.

RPE replacement therapy using stem cells has long been investigated as a therapeutic option for age‐related macular degeneration (AMD)^[^ ^5^, ^36^ ^]^ and genetic retinal diseases such as Stargardt disease.^[^ ^9^ ^]^ In both cases, the macular RPE atrophy is followed by photoreceptor cell death. In addition, RPE patches could be useful in “wet” AMD, aiming to couple intact RPE to photoreceptors displaced due to the fibrovasculature overgrowth.^[^ ^37^ ^]^

Validation of clinical‐grade protocols across laboratories worldwide is paramount for establishing standardized and reproducible methodologies, ensuring consistent and reliable outcomes in therapeutic interventions, thereby fostering global collaboration and propelling the field of regenerative medicine forward. Additionally, the validation of transplantation in larger animal models such as pigs enables researchers to evaluate the scalability and feasibility of the procedures for potential clinical translation. By demonstrating the efficacy and safety of these protocols in animals that closely resemble human physiology, researchers can fortify the evidence base for regulatory approval and eventual clinical trials in human patients.

Our study further underscores the utility of the pig model for advancements in retinal cell therapy. The decision to transplant hiPSC‐RPE patches onto healthy RPE in minipigs in our study was intentional. By doing so, we evaluated key aspects of our approach—cell survival, integration, and compatibility—in a controlled environment without the additional complexities of retinal degeneration. That's why the cells are put on top of healthy pig´s RPEs, mimicking the condition of patients with dysfunctional but structurally preserved RPE, which is a relevant scenario for our proposed therapeutic strategy. Additionally, allogeneic transplantation is designed to address a wide range of retinal diseases beyond those characterized by macular degeneration. In diseases like Stargardt and Best disease, a structurally preserved host RPE structure is critical for transplanted cells to integrate effectively and restore function, making our strategy particularly relevant. Testing our patches on healthy retina ensures that the treatment is safe and broadly applicable, laying the groundwork for future studies in disease‐specific models. These subsequent studies will explore the therapeutic efficacy in degenerative conditions, allowing for a more targeted approach to macular degeneration and other disorders. The next phase of our research involves testing the patch efficacy in pathological conditions by replicating AMD features through laser‐induced injury of the host RPE in the visual streak or by utilizing minipig genetic models

## 4 Experimental Section

### Maintenance of hiPSCs

The cell line employed in this study included previously generated hiPSCs derived from a healthy subject (Ctrl3‐FiPS4F1) previously generated in our laboratory.^[^ ^24^ ^]^

Cells were maintained on human VITRONECTIN in E8 medium (StemCell Technologies). The cell medium was changed daily and enzymatically passaging with dispase (1 mg mL^−1^, StemCell Technologies) every five to seven days at splitting ratios of 1, 6 to 1, 15.

### RPE Differentiation and Culture

To derive RPE from hiPSC, a previously established clinical‐grade protocol was employed, incorporating minor modifications for optimization^[^ ^18^ ^]^ (Figure 1A). Following 40 days of differentiation, the cells were cultured on poly‐L‐lactide‐*co*‐D,L‐lactide (PDLLA) scaffolds coated with human laminin and sustained for eight weeks in RPE medium, with medium changes occurring every other day (Figure 1A). The nanofibrous membrane, composed of randomly assembled fibers forming the scaffold, was prepared using solution electrospinning.^[^ ^17^ ^]^ The developed scaffold boasts distinct advantages over others, including significantly higher porosity (exceeding 70%), reduced thickness, and enhanced degradability.^[^ ^17^ ^]^ Three forms of scaffolds were fabricated, frameless scaffolds and scaffolds with three inserted circular frames (OD 3.5 mm) for testing of cell culture, and scaffolds with oval frames for surgical transplantation. The 30 µm‐wide frames were precisely cut using a femtosecond laser from bi‐axially oriented 36 µm thick polyethylene terephthalate (PET) foils. Following cell cultivation on the scaffolds, samples were obtained using a 4 mm biopsy punch from the scaffolds with three inserted circular frames, preserving their flat shape and facilitating more effective biological testing. In summary, identical scaffolds were employed, incorporating frames to significantly streamline the process of manipulation for immunocytochemical analysis.

### Transmission Electron Microscopy

The hiPSC‐RPE Patches for electron microscopy were cultured in parallel to patches for transplantation and at the time of transplantation underwent fixation in a solution comprising 4% paraformaldehyde and 2% glutaraldehyde in 0.1 m sodium phosphate buffer (pH 7.2–7.4) for a duration of 2 h. Subsequently, the fixed patches were rinsed with the same buffer and post‐fixed in a 1% OsO4 solution in phosphate buffer. After gradual dehydration using an ethanol series, the patches were embedded in EPON 812 for sectioning.

Semi‐thin sections were stained with 1% toluidine blue in 3% sodium tetra‐borate and examined using a Leica DMR light microscope (Leica Microsystems). For more in‐depth analysis, both semi‐thin and ultrathin sections were obtained using a Leica Ultracut R ultramicrotome (Leica Microsystems).

The ultrathin sections were further stained with lead citrate and uranyl acetate before being scrutinized under a JEM‐1400 Plus electron microscope (JEOL GmbH, München, Germany) to discern the ultrastructural details of the RPE patches.

### RNA Extraction and Quantitative Real‐Time PCR (qRT‐PCR)

Batches of cells cultured in parallel to the transplantation patches Cells were harvested via centrifugation, and total RNA was extracted using the RNeasy Mini Kit (Qiagen, Hilden, Germany) following the provided protocol. To eliminate any genomic DNA contamination, the isolated RNA underwent DNase1 treatment. The QuantiTect Reverse Transcription Kit (Qiagen) was employed to synthesize cDNA from 1 µg of total RNA following the manufacturer's instructions.

For quantitative real‐time PCR (qRT‐PCR), relative quantification analysis was conducted using the LightCycler 480 System (Roche). The PCR cycling program involved an initial denaturation step at 95 °C for 10 min, followed by 40 cycles of denaturation at 95 °C for 15 s and annealing/elongation at 60 °C for 1 min. The reactions were carried out in triplicate using TaqMan Gene Expression Master Mix. The list of TaqMan probes utilized (Applied Biosystems, Foster City, CA, USA) could be provided under request.

### Immunocytochemistry

Human iPSC‐RPE cells in parallel patches underwent a sequence of preparatory steps for immunofluorescence staining, starting with a PBS wash followed by fixation in 4% paraformaldehyde for 15 min. Post‐fixation, cells were subjected to two PBS washes and then immersed in a blocking solution (composed of 3% normal goat serum and 0.5% Triton‐X100 in PBS) for 1 h at room temperature to prevent nonspecific binding.

Subsequently, the cells were incubated overnight at 4 °C with the primary antibody. The next day, three to five PBS washes were performed to eliminate any unbound primary antibody. Following this, cells were incubated with an appropriate secondary antibody (diluted at 1, 500, Invitrogen). After the secondary antibody incubation, cell nuclei were stained with 4′,6‐diamidino‐2‐phenylindole dihydrochloride (DAPI) and washed thrice in PBS. For samples cultivated on nanofibrous scaffolds, VectaShield Mounting Medium (Vector Lab, Burlingame, CA, USA) was employed for mounting. Immunofluorescence images were captured using a Leica confocal microscope TCS SP8 with an HCX PL APO lambda blue 63X/1.4 OIL objective, and Z‐stacks were analyzed to demonstrate polarized expression.

Specific antibodies used for immunostaining are detailed in Table S1 (Supporting Information).

### Scaffold Preparation

Scaffolds with embedded supporting frames were prepared as described previously.^[^ ^15^ ^]^ Briefly, nanofibrous membranes composed of 380 nm fibers were prepared by electrospinning of poly (L‐lactide‐*co*‐D, L‐ lactide) in pyridine (11 wt.%) with the addition of formic acid (2.2 µL g^−1^ of polymer solution). The area density of the prepared scaffolds was 130 µg cm^−2^ (standard deviation, SD 10 µg cm^−2^), porosity 72%, pore dimension 0.4 µm (SD 0.2 µm) and thickness measured by surface profiler 3.7 µm. Tiny 30 µm wide oval supporting frames were obtained by cutting poly(ethylene terephthalate)‐based foil of thickness 36 µm using a femtosecond laser microfabrication station. The resulting membrane with the incorporated frame was fixed to the body of the cell‐cultivation cup Falcon.

### Animals Used in the Experiments

Nine females and five males (22–35 kg B.W., 9–10 months old) of Libechov minipigs originating from the breeding station of the Institute of Animal Physiology and Genetics in Libechov, Czech Republic (CZ) were used in the study. They were housed in groups of two or were kept individually. All experiments were carried out according to the guidelines for the care and use of experimental animals and approved by the Resort Professional Commission of the Czech Academy of Sciences for Approval of Projects of Experiments on Animals (protocol numbers 64/2019).

### Surgical Procedure

After intramuscular premedication of experimental animals with “TKX mixture” containing tiletamine 4 mg kg^−1^ + zolazepam 4 mg kg^−1^ (Zoletil 100, Virbac, France), ketamine 5 mg kg^−1^ (Narketan 10, Chassot, Switzerland), and xylazine 1 mg kg^−1^ (Rometar 2%, Spofa, CZ), general anesthesia was induced and maintained by intravenous application of 1% Propofol (FRESENIUS PROPOFOL 1% EMULSION) and Rocuronium bromide (25 mg) by infusion pump (30–40 mL h^−1^). Analgesia of animals was achieved by intramuscular application of Flunixine Meglumine (Flunixin Injection, Norbrook, 2 mL/45 kg B.W.) and Tramadol (Tramal, STADA Arzneimittel AG, Bad Vilbel, 100 mg/animal) during the surgery. In addition, a single injection of depot methylprednisolone (DEPO‐MEDROL, 80 mg/animal) and depot Penicillin/Streptomycin (Shotapen, Virbac SA, 1 mL/10 kg B.W.) was applied during implantation.

The scaffold with seeded hiPSC‐RPE cells for implantation (the oval‐shaped cell graft with dimension 5.2 mm × 2.1 mm) was cut from the cell cultivation insert by modified biopsy punch. The cell graft was delivered to the subretinal space by a standard lens‐sparing three‐port pars plana vitrectomy using an in‐house implantation injector as described in the previous paper (14)

### Noninvasive Examinations of Implanted Eyes (OCT, Fundus Camera)

The eyes with the implanted carriers were investigated using color non‐mydriatic fundus camera iCam (Optovue, Fremont, CA, USA) and the spectral‐domain OCT iVue (Optovue, Fremont, CA, USA). A lid speculum was used to maintain open eyes. To keep the ocular surface moist and to obtain a better OCT image the cornea of the animal was washed with saline solution (0.9% NaCl) every 30–60 s. For optimal focusing of the implant on the fundus, the infrared reflective light of the OCT device was employed.

### Immunosuppression

Five to seven days before cell‐seeded scaffold implantation, the first dose of tacrolimus‐loaded polymer microspheres (TLPM) (15) containing tacrolimus dose 0.8 mg kg^−1^ B.W was subcutaneously (behind the ear) administered. White powder of TLPM was diluted in sterile saline (cca 3 mL/animal) before application by 5 mL Luer Lock syringe and BD 16G needle as a white suspension. Every 12th day the TLPM was administered to animals to maintain the tacrolimus blood concentration of animals ≈20–30 ng mL^−1^. Minipig´s tacrolimus blood concentration was detected on the day of cell‐seeded scaffold implantation and before every next TLPM application (Tacrolimus dose ranged from 0.3 to 0.8 mg kg^−1^ B.W.). Importantly on the day of cell‐seeded scaffold implantation, the single injection of depo‐medrol (80 mg/animal) was intramuscularly injected without next reinjection.

### Multiplex Analysis of Cytokines

For analysis of cytokine profiles in porcine blood plasma, MILLIPLEX MAP Porcine Cytokine/Chemokine Magnetic Bead Panel (cat. no. PCYTMG‐23K‐13PX, Merck Millipore, USA) was used, enabling the simultaneous measurement of 13 proteins – namely IL‐1α, IL‐1β, IL‐1RA, IL‐2, IL‐4, IL‐6, IL‐8, IL‐10, IL‐12, IL‐18, GM‐CSF, IFNγ, and TNF‐α. Plasma samples were thawed on ice and pre‐cleaned by centrifugation at 16 000 x g for 10 min at 4 °C. All samples, standards, and backgrounds were assayed in 2 technical replicates. The assay was performed as previously described.^[^ ^38^ ^]^ Standard curves were extended at the lower end by additional dilutions of calibration standards. The Luminex instrument system was properly calibrated according to the manufacturer's instructions. The fluorescence intensities of at least 50 microspheres of each analyte per sample were acquired on Luminex 200 analyzer with xPonent software (version 3.1.871.0) (Luminex Corp., USA), and the median fluorescence intensity (MFI) was used for quantitation of cytokine concentrations. Raw MFI data were exported from xPonent software in csv. Format and analyzed in the Belysa software package v1.2.2 (Merck Millipore, USA). Standard curves were fit using 4‐ or 5‐parameter logistic regression.

Statistical analysis was performed using Instat 3 software (GraphPad Software, USA). Non‐parametric Kruskal Wallis test followed by Dunn´s multiple comparison was employed.

### Euthanasia and Enucleation

Euthanasia was performed under general anesthesia consisting of the intramuscular injection of the “TKX mixture” and the intravenous overdosing of 1% Propofol (FRESENIUS PROPOFOL 1% EMULSION, 24 mL/animal) followed by exsanguination. Before whole eye bulbs were removed from the orbit the nasal side (at the corner of the eye) of the eyeball was marked with a surgical suture in the area of the limbus. Then enucleated eyes were fixed in 4% paraformaldehyde (PFA) at 4 °C for 24 h before being stored in PBS at 4 °C before further processing.

### Processing of Eyecups

The anterior cup of the eye was removed by circumferential incision at the limbus. The eyecup was oriented according to the surgical suture and the pig's visible characteristic major blood vessels, and the implanted scaffold was identified in the nasal central retina, and isolated with the sclera attached. Control eyes were dissected the same way, and the nasal central retina was collected. All tissues were cryoprotected in graded sucrose solutions as described in detail.^[^ ^16^ ^]^ Vertical or tangential sections (14 m) were cut with a cryomicrotome (SLEE Medical GmbH, Mainz, Germany) and collected on Superfrost slides.

### Immunohistochemistry

For frozen sectioning, porcine eyecups were treated as previously described.^[^ ^16^ ^]^ Briefly, immunostaining was performed by employing the two‐step indirect method. Sections were incubated at room temperature overnight in primary antibodies (Table S1, Supporting Information). Immunofluorescence was performed using Alexa Fluor 488‐conjugated secondary antibodies (#21202, Thermo Fisher Scientific, Bremen, Germany) or Alexa 594 (#21207, Thermo Fisher Scientific, Germany) and Alexa 647 (# A‐21447, Thermo Fisher Scientific, Germany).

### Laser Scanning Confocal Microscopy

Confocal images were captured using an Olympus FV10i confocal microscope, equipped with Argon and HeNe lasers. A high‐resolution scanning of image stacks was performed with a UPlanSApo X 60/1.35 (Olympus) oil‐immersion objective at 1024 × 1024 pixels and a z‐axis increment of 0.3 µm. For an analysis of the immunolabelled cells and their processes, a stack of 2–12 sections was used (0.7 µm z‐axis step size). Cell processes were reconstructed by collapsing the stacks into a single plane. The brightness and contrast of the final images were adjusted using Adobe Photoshop CS5 (San Jose, CA, USA).

### Statistical Methods

The data were presented as mean ± SEM of at least three biological replicates. Statistical analysis was conducted using an unpaired *t*‐test to determine differences in gene expression. This analysis was performed using GraphPad Prism 6 software (GraphPad Software). Significant differences were indicated when the p‐value was less than 0.05 (^*^ *p* ≤ 0.05, ^**^ *p* ≤ 0.01, ^***^ *p* ≤ 0.001, ^****^ *p* ≤ 0.0001).

## Conflict of Interest

The authors declare no conflict of interest.

## Supporting information



Supporting Information

## Data Availability

Data sharing is not applicable to this article as no new data were created or analyzed in this study
