# Supplementary material for: Delivery of Human iPSC‐Derived RPE Cells in Healthy Minipig Retina Results in Interaction Between Photoreceptors and Transplanted Cells
Source: Adv Sci (Weinh). 2025 Apr 2;12(20):2412301. doi: 10.1002/advs.202412301 (PMC12120741; doi:10.1002/advs.202412301)
Supplement: Supplementary file 1 — Supporting Information [file ADVS-12-2412301-s001.docx]

**Supplementary Figures**


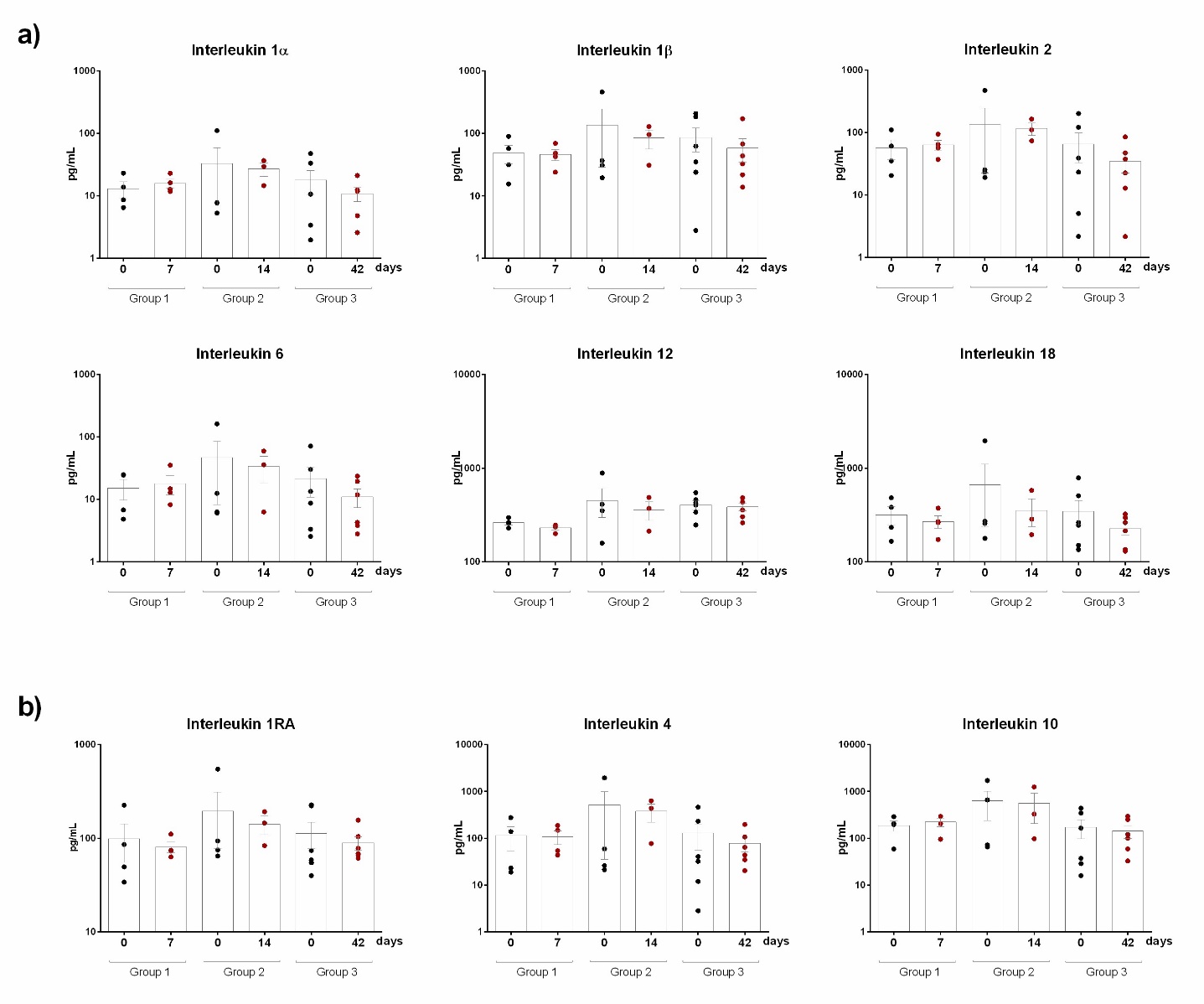


***Figure S1. Interleukin concentration in vivo data from all sets of animals is based on the group, which is based on the day of sacrifice.*** *No significant statistical difference observed in the level of interleukins, which suggests there was no significant immune response present in the operated eye. Group 1 (0 – 7 D), Group 2 (0 – 14 D), Group 3 (0 – 28 D). Minimum n=3/group.*


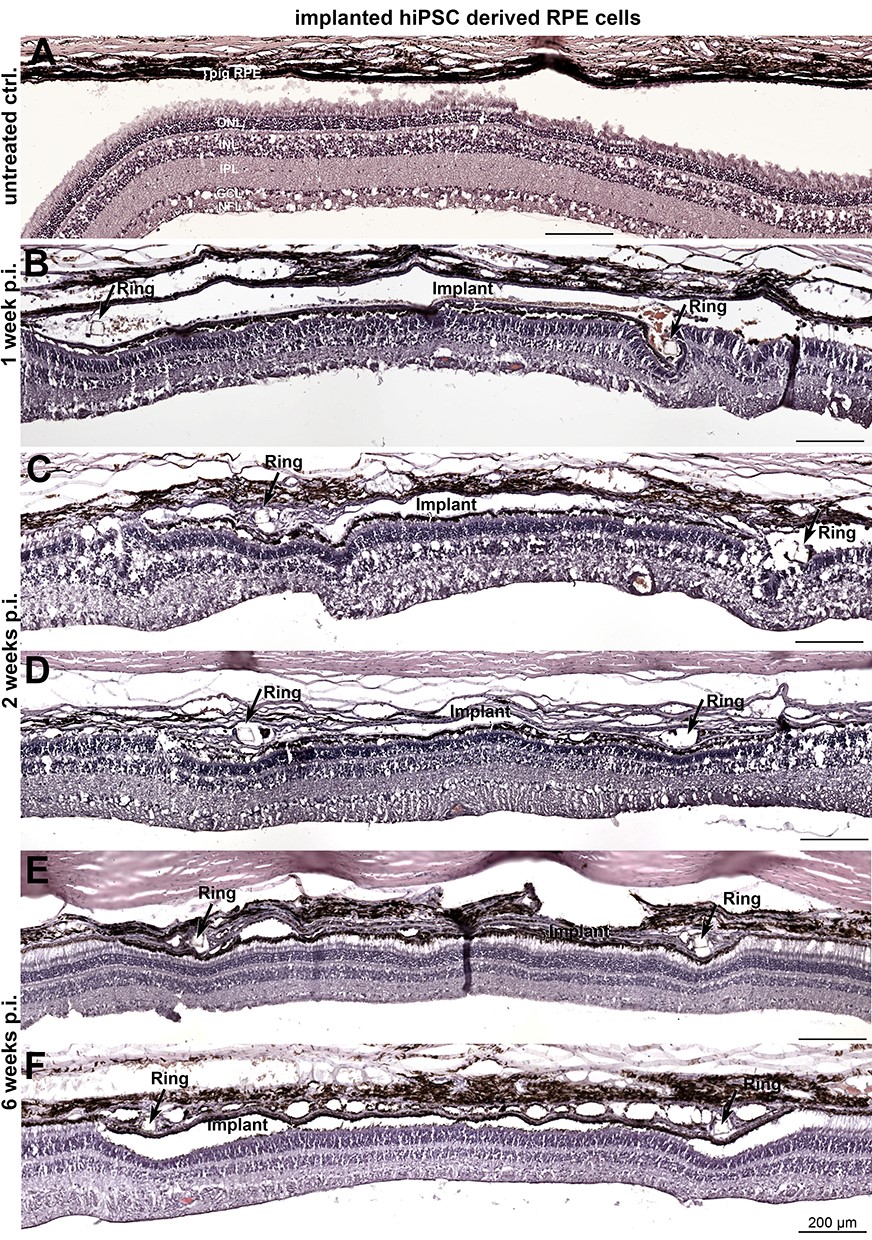


***Figiure S2: Hematoxylin & eosin staining of the retinal area containing the transplanted cells on nanofibrous carrier membrane surrounded by PET ring structure (arrows).*** *Examples of all investigated minipigs are included. Observations are presented at 1 week (upper two rows), 2 weeks (middle three rows), and 6 weeks (bottom three rows). Implanted hiPSC-RPE cells are heavily pigmented and closely connected with photoreceptor inner and outer segments in most individuals. In some eyes (C, G) little connection between hiPSC-RPE cells and PhR resulted in thinning of the retina. Carrier dimensions: 5.2 mm x 2.1 mm. ONL = outer nuclear layer; INL = inner nuclear layer; IPL = inner plexiform layer; GCL = ganglion cell layer; NFL = nerve fiber layer. All scale bars represent 200 µm.*


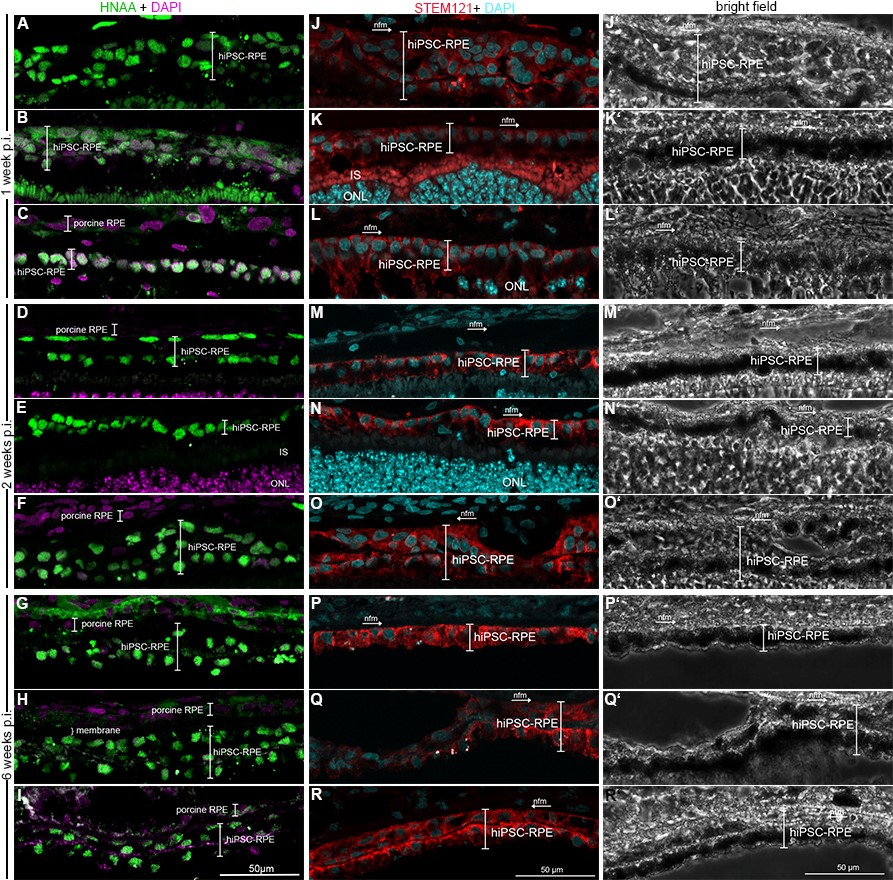


***Figure S3 :*** ***Immunofluorescence of RPE cell markers.*** *Bestrophin (green) (****A-D****) and cellular retinaldehyde binding protein (CRALBP, red) (****E-H****)with the respective antibodies in the minipig retina holding the nanofibrous carrier membrane with hiPSC-RPEcells followed up to 6 weeks. (A,E) are images of the control eyes to demonstrate specificity of the staining. Immunofluorescence analysis are presented at 1 (B, F), 2 (C, G), and 6 (D, H) weeks p.i.. Nuclear staining by DAPI is shown in magenta in (A–D) and blue in (E–H)). ONL = outer nuclear layer, INL = inner nuclear layer, IS = inner segment.*


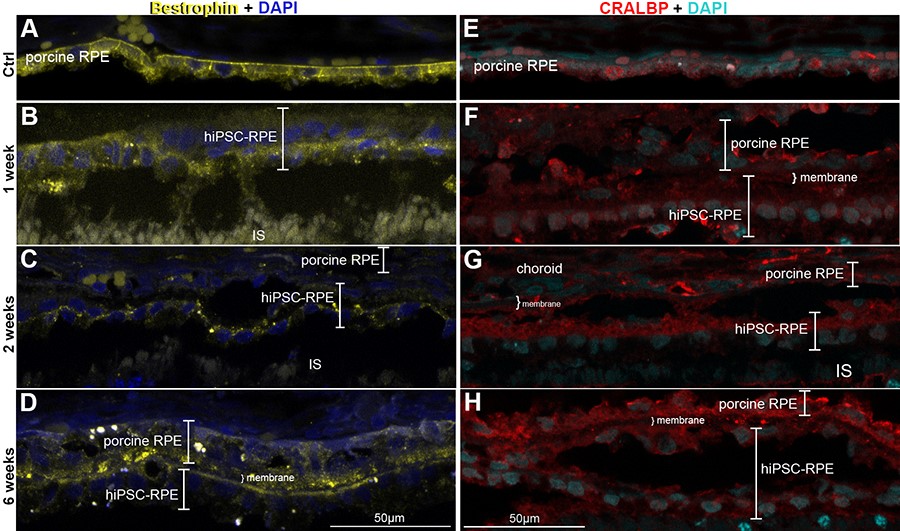


***Figure S4: Expression of Human Nuclear Antigen (HNAA, green) and STEM121 (red) on hRPEs implanted on nanofibrous carriers, followed up to 6 weeks****. Examples of all investigated minipigs are included. Tissue was stained with HNAA (A–I) and STEM121 (J-R) antibodies. The third panel (J’-R’) shows bright field images of the STEM121 staining. Observations are presented at 1 week (upper three rows), 2 weeks (middle three rows), and 6 weeks (bottom three rows). The implanted hRPE cells appear as a monolayered or a multi-layered cell structure after 1, 2, or 6 weeks showing a high cell density. i.e. the distance between the nuclei is very small which is unusual for RPE cells. Nuclear staining was performed by DAPI (purple and light blue). IS = inner segments; ONL = outer nuclear layer; nfm = nanofibrous membrane; hiPSC-RPE = human iPSC derived RPE cells; RPE = retinal pigment epithelium.*


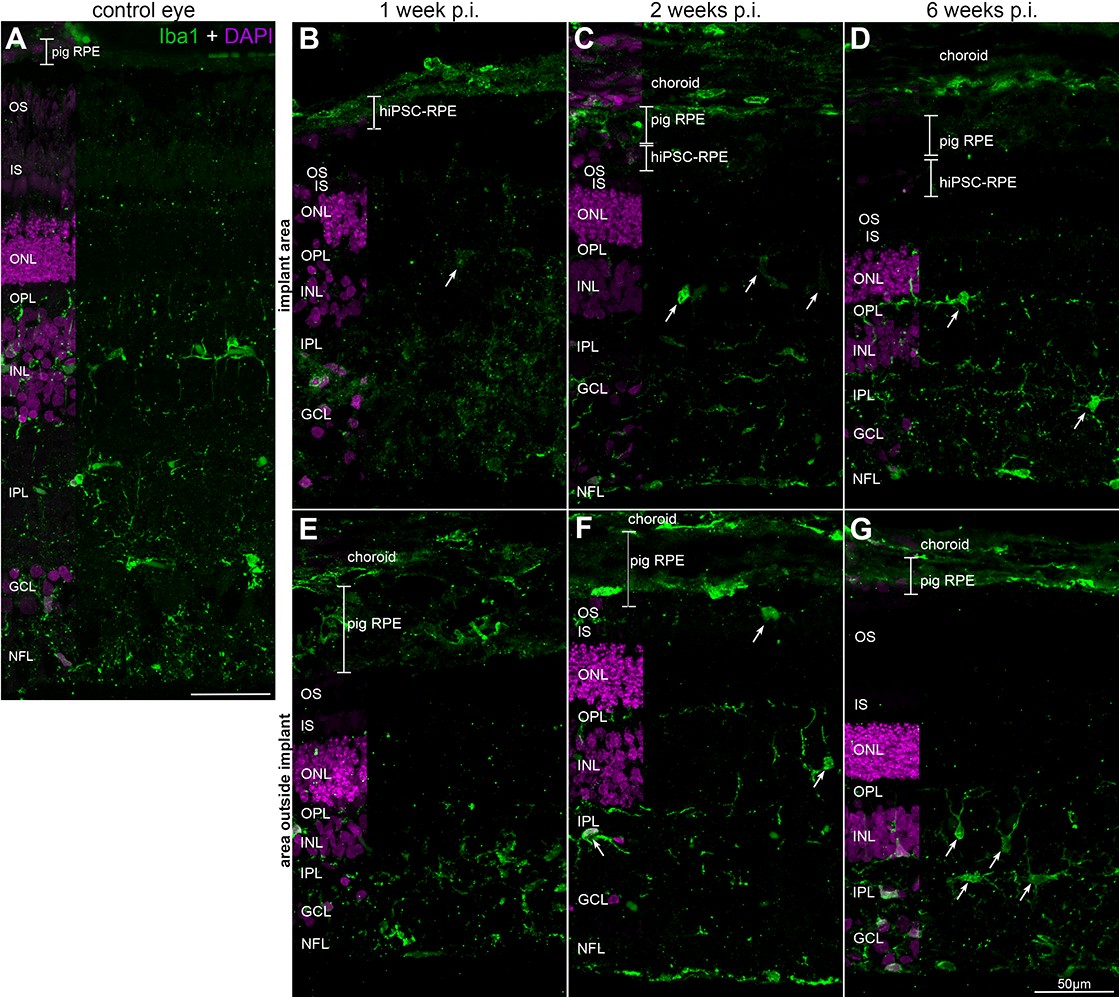


***Figure S5: Immunoreactivity of microglia marker Iba1 in vertical sections of minipig retina inside (B- D) and outside (E–G) the implant area and control eye (A).*** *There is little difference in iba1-* *immunoreactivity between the respective retinal areas. In 1 week post-implantation (B, E) no distinct* *iba1-immunoreactive microglia cells are visible. Only in the inner plexiform and ganglion cell layer* *dot-like rough processes are obvious. After 2 weeks (C, F) individual microglia somata show iba1* *immunoreactivity (arrows). Especially outside the implant area, more microglia cells with their* *processes are visible. 6 weeks post-implantation (D, G) iba1-immunoreactive cells are present in the* *inner and outer plexiform layer with their processes ramifying into the nuclear layers in a* *characteristic manner like in untreated minipig eyes (A). Dot-like iba1 immunohistochemistry occurs* *at all time points shown here. IS = inner segment, ONL = outer nuclear layer, OPL = outer plexiform* *layer, INL = inner nuclear layer, IPL = inner plexiform layer, GCL = ganglion cell layer, NFL = nerve fiber* *layer. Scale bars are 50 µm for all micrographs.*

**Tables**

**Table S1: Antibodies used for immunohistochemical analysis.**

| **Protein** | **Source** | **Manufacturer** | **Working Dilution** |
| --- | --- | --- | --- |
| **HNAA** | **mAb mouse** | **Novus Biologicals, UK, Abingdon, (235-1)**  **NBP2-34342-0.1 mg** | **1:300** |
| **STEM121** | **mAb mouse** | **Takara Bio Inc., Kusatsu, Japan, cat.**  **No. Y40410** | **1:1000** |
| **Bestrophin** | **mAb mouse** | **Santa Cruz Inc., Germany, Heidelberg, cat.**  **No. sc-32792** | **1:50** |
| **CRALBP** | **mAb mouse** | **Novus Biologicals UK, Abingdon, (B2)**  **cat. No. NB100-74392** | **1:100** |
| **ZO-1** | **mAb mouse** | **ThermoFisher Scientific, USA, Cat. No. 33-9100, Lot Number: SH255451** | **1:100** |
| **Vimentin** | **mAb mouse** | **Cell Signaling, (D21H3), USA, cat. No. 5741** | **1:100** |
| **GFAP** | **pAb rabbit** | **Merck Millipore, Germany, Darmstadt,**  **cat. No. AB5804, lot No. 2464502** | **1:1000** |
| **IbaI, Microglial Marker** | **mAb mouse** | **Abcam, Cambridge, UK,**  **cat. No. ab15690, clone 1022-5** | **1:250** |
| **Lectin PNA Conjugate Alexa Fluor 488** |  | **Molecular probes cat. No. L-21409** | **1:300** |
